# Passive Oscillatory Body Motion Attenuates Pain‐Related Behaviors and Increases β‐Endorphin Immunoreactivity in a Murine Fracture Model: A Preliminary Study

**DOI:** 10.1002/brb3.71614

**Published:** 2026-07-23

**Authors:** Ryoma Kato, Kazuhiro Hayashi, Yuri Okada, Rina Okura, Fuyu Niida, Jun Miyata, Naoyoshi Sakitani, Yasuhiro Sawada, Akira Ito

**Affiliations:** ^1^ Department of Advanced Physical Therapy, Human Health Sciences, Graduate School of Medicine Kyoto University Kyoto Japan; ^2^ Integrated Research Center For Self‐Care Technology, National Institute of Advanced Industrial Science and Technology (AIST) Takamatsu Japan; ^3^ Health and Medical Research Institute, National Institute of Advanced Industrial Science and Technology (AIST) Takamatsu Japan; ^4^ Department of Clinical Research National Rehabilitation Center For Persons with Disabilities Tokorozawa Japan

**Keywords:** β‐endorphin, exercise‐mimetic body motion, hypothalamus–PAG pathway, murine fracture model, periaqueductal gray

## Abstract

**Objective:**

This preliminary study aimed to investigate whether passive oscillatory body motion (PBM), a mechanical stimulus that reproduces vertical accelerations comparable to those generated during treadmill running, could activate the β‐endorphin (β‐END)–related hypothalamus–periaqueductal gray (PAG) pathway and alleviate fracture‐induced pain.

**Methods:**

A murine fracture model was established in C57BL/6J mice by inducing a left tibial fracture followed by 3 weeks of immobilization. Mice were randomly divided into a PBM group and a control group, and PBM was initiated the day after fracture (±5 mm amplitude, 2 Hz, 30 min/day, 5 days/week, for 3 weeks). To measure pain thresholds, paw withdrawal thresholds were measured in an unblinded manner using the von Frey filament test. β‐END immunoreactivity and opioid receptor mu 1 (*Oprm1*) mRNA in the PAG, as well as pro‐opiomelanocortin (*Pomc*) mRNA expression in the hypothalamus, were analyzed by immunofluorescence and qPCR, respectively.

**Results:**

PBM significantly increased the paw withdrawal threshold ratio (*p* < 0.001) and β‐END immunoreactivity (*p* = 0.02) in the PAG. Moreover, as an exploratory observation, *Pomc* mRNA expression tended to increase in the PBM group (*p* = 0.07), whereas *Oprm1* mRNA expression did not differ between the two groups (*p* = 0.69).

**Conclusion:**

This preliminary study provides initial evidence that PBM may attenuate fracture‐induced pain‐related behaviors and increase β‐END immunoreactivity in the PAG, possibly via the hypothalamus–PAG pathway. PBM may represent a potential exercise‐mimetic approach for pain relief, although further studies are needed to confirm its efficacy and underlying mechanisms.

## Graphical abstract

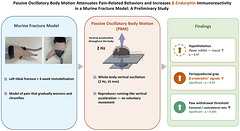

## 1 Introduction

Pain is one of the major factors leading to impaired motor function, reduced physical activity, and decreased quality of life (QOL). Despite advancements in pain management strategies, treating chronic pain remains a significant challenge. A questionnaire survey conducted in Japan reported that approximately 40% of the 2628 respondents (aged 20–99 years) experienced chronic pain (Inoue et al. 2015). One of the factors contributing to pain chronification is the development of fear‐avoidance behavior following an acute episode (Fujii et al. 2013). In this fear‐avoidance model, the experience of pain triggers catastrophic thinking and subsequent activity avoidance, creating a vicious cycle that promotes chronicity (Vlaeyen and Linton 2000). Consequently, early intervention after pain onset is recommended to prevent this progression (Gatchel et al. 2003; Lalchun and Duff 2025).

Exercise therapy is widely used as a non‐pharmacological approach for pain relief, largely mediated by the activation of the endogenous opioid system. β‐endorphin (β‐END), an endogenous opioid peptide, is released in response to exercise and exerts analgesic effects (Bender et al. 2007; Koltyn 2000; Xue et al. 2020). β‐END is synthesized in pro‐opiomelanocortin (*POMC*) neurons within the hypothalamic arcuate nucleus (Kang et al. 2021; Tao et al. 2023). These neurons project their axons to central regions such as the periaqueductal gray (PAG) (Bagley and Ingram 2020; Ossipov et al. 2014), where β‐END acts on μ‐opioid receptors (MOR) to activate the descending pain modulatory system and thereby produce analgesia (Fields 2004; Heinricher et al. 2009). However, severe pain often precludes patients from performing voluntary exercise, thereby limiting the applicability of exercise therapy. This background highlights the need for alternative strategies that can deliver exercise‐mimetic stimulation without requiring active physical movement.

Recent studies indicate that mechanical stimulation mimicking body motion may regulate physiological functions. We previously demonstrated that vertical head oscillation induces interstitial fluid flow and modulates angiotensin II receptor signaling in the brainstem, thereby reducing blood pressure in hypertensive rats (Murase et al. 2023). These findings suggest that passive mechanical stimulation, such as passive head motion (PHM), which reproduces acceleration stimuli similar to those generated during running, may evoke exercise‐mimetic physiological responses.

**FIGURE 1:**
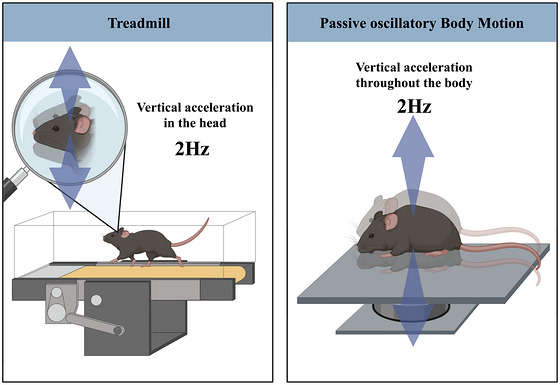
Schematic diagram illustrating passive oscillatory body motion (PBM) as an exercise‐mimetic mechanical stimulus. PBM delivers vertical oscillatory stimulation at 2 Hz throughout the body of the mouse. The frequency of 2 Hz was chosen because it reproduces vertical acceleration waveforms in the head during treadmill running in mice. Vertical head oscillation has been reported to provide mechanical stimulation to brain cells via changes in interstitial fluid dynamics, thereby modulating cell‐surface receptor expression (Murase et al. 2023). This figure was created in BioRender. Kato R. (2026) https://BioRender.com/6kla12a.

Passive oscillatory body motion (PBM), similar to PHM, provides vertical acceleration stimuli to the body. PBM offers the advantage of reproducing passive mechanical stimuli comparable to those generated during treadmill running, even when active exercise is unfeasible (Figure 1). While several mechanisms for treadmill‐induced β‐END release have been reported (Kang et al. 2021), it remains unclear whether the vertical acceleration waveforms themselves contribute to these effects. Elucidating this relationship may reveal a previously unrecognized mechanism underlying exercise‐induced β‐END release. Therefore, this study was designed as a preliminary investigation to provide initial evidence of the analgesic effects of PBM. We further explored the underlying central mechanisms by assessing pain‐related behaviors, hypothalamic *Pomc* mRNA expression, and *Oprm1* mRNA expression, which encodes the MOR, as well as β‐END immunoreactivity in the PAG, using a murine fracture model that recapitulates clinically relevant challenges of reduced activity and pain chronification following bone injury.

## 2 Methods

### 2.1 Experimental Animals

C57BL/6J mice (8 weeks old; male 20–24 g, female 15–18 g; Shimizu Laboratory Supplies Co., Ltd., Kyoto, Japan) were used in this study. Animals were housed under controlled conditions (22 ± 2°C, 12 h light/dark cycle) with free access to water and standard chow. All experimental procedures were approved by the Animal Research Committee of Kyoto University (Approval No. MedKyo24557).

### 2.2 Establishment of a Murine Fracture Model

**FIGURE 2:**
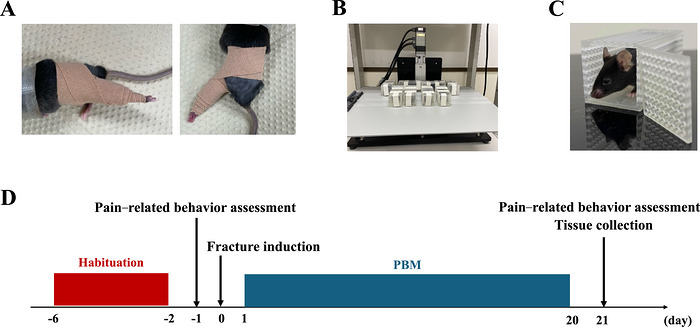
Experimental setup and protocol for the murine fracture model and passive oscillatory body motion (PBM) intervention. (A) Representative photograph of the murine fracture model with cast immobilization; (B) vertical linear motion device used to deliver passive oscillatory body motion (PBM); (C) mouse chamber mounted on the motion device; and (D) experimental timeline and protocol.

The murine fracture model was established based on a previous report with minor modifications (Shi et al. 2018). Mice were anesthetized via intraperitoneal administration of a mixture consisting medetomidine (0.75 mg/kg), midazolam (4.0 mg/kg), and butorphanol (5.0 mg/kg). Under anesthesia, a transverse fracture was manually induced at the mid‐shaft of the left tibia of each mouse. To minimize inter‐experimental variability, all fracture procedures were performed by a single experimentalist (RK) throughout the study, ensuring procedural consistency (Supplementary Figure 1). Following fracture induction, the left hind limb was immobilized for 3 weeks using a cylindrical cast (Gips Plus One, Alcare Co., Ltd., Tokyo, Japan), secured with kinesiology tape (C&G Systems Co., Ltd., Tokyo, Japan) (Figure 2A). PBM intervention was applied during this immobilization period to examine its effect on pain progression. Casts and tapes were remade or replaced as needed (0–2 times per week), depending on the degree of loosening. Recasting was performed under isoflurane inhalation anesthesia (3–5%) for 3–5 min per mouse. Animals in which the cast or tape became detached were excluded from analysis.

### 2.3 Group Allocation and PBM Intervention

Six days before fracture induction, mice were randomly assigned to either the PBM group (*n* = 16; 8 males and 8 females) or the control group (*n* = 16; 8 males and 8 females). One mouse in the PBM group that died during the housing period was excluded from the analysis.

Instead of the PHM used in previous studies to vertically oscillate the heads of rodents (Murase et al. 2023; Ryu et al. 2020), we applied PBM. This approach does not require anesthesia, thereby eliminating potential confounding effects of anesthesia on pain and inflammation (Matta et al. 2008). Vertical oscillation with a peak‐to‐peak amplitude of 10 mm at 2 Hz was generated using a vertical linear motion system (V100‐VL; Tsubosaka Electric Co., Ltd., Tokyo, Japan) (Figure 2B), producing peak vertical accelerations of approximately 0.3 × *g*. These PBM parameters (2 Hz ± 5 mm amplitude) were selected based on a previous study to mimic the vertical acceleration experienced during treadmill running (Ryu et al. 2020). This magnitude was modestly lower than the accelerations (≥ 0.5 × *g*) previously shown to elicit anti‐hallucinogenic and anti‐hypertensive effects (Murase et al. 2023; Ryu et al. 2020), which were achieved using impulsive waveforms. Our previous simulations (Murase et al. 2023) indicated that sinusoidal oscillation could provide comparable efficacy with reduced peak acceleration. Notably, this approach enabled stable whole‐body oscillation of loosely restrained, awake mice.

During PBM intervention, mice were restrained in a chamber (Figure 2C). Although they were able to change their head‐to‐tail orientation within the chamber, the mice remained calm and exhibited no escape behavior throughout the 30 min session.

Mice were acclimated to the restraint and device environment during the week preceding the fracture induction. During the habituation period, the duration of PBM exposure was gradually increased: 10 min on day 1, 20 min on day 2, and 30 min on days 3–5. Control mice were restrained for the same duration but did not receive PBM.

PBM intervention was initiated one day after fracture induction and administered for 30 min/day, 5 days/week, for three consecutive weeks (Figure 2D). This 3‐week duration corresponds to the typical healing period associated with hard callus formation in this murine model (Manigrasso and O'Connor 2004). Furthermore, the fixation procedure used here has been established to produce a murine model in which pain gradually worsens and undergoes chronification over time (Shi et al. 2018). The intervention schedule (30 min/day, 5 days/week) was selected based on previous studies. The session duration (30 min/day) was based on a protocol showing that mechanical oscillation induces physiological changes in neurons (Ryu et al. 2020). The intervention frequency of 5 days per week was selected based on an exercise protocol shown to increase β‐END levels and elevate pain thresholds in a murine pain model (Stagg et al. 2011). Control mice were also placed in a mouse chamber (Figure 2C) in a prone position on a static platform. Importantly, restraint alone did not affect pain sensitivity, as confirmed in preliminary experiments (Supplementary Figure 2). In both groups, the chamber was covered with an opaque shroud during the intervention to block visual stimuli.

### 2.4 Pain–Related Behavior Assessment

Mechanical hypersensitivity was assessed by the von Frey filament test (Martinov et al. 2013) using an electronic von Frey apparatus (Bioseb, Vitrolles, France). Mechanical stimuli were applied to the plantar surface of both hind limbs to measure paw withdrawal thresholds (g). All measurements were performed by a single investigator who was not blinded to the experimental groups. In each session, three measurements were obtained from each paw, and the mean value was used for analysis. To minimize inter‐individual variability in mechanical sensitivity, the ratio of thresholds (fractured side/contralateral side) was calculated as an index of pain sensitivity. Assessments were performed twice: before fracture induction and after cast removal (3 weeks post‐induction). The final assessment was performed approximately 14–19 h after the last intervention.

### 2.5 Immunohistochemistry

Immunohistochemistry was performed to evaluate β‐END immunoreactivity in the PAG. At 3 weeks post‐fracture, brains were collected from 8 mice in the PBM group and 8 mice in the control group. The brains were fixed overnight in 4% paraformaldehyde at 4°C, followed by cryoprotection in 20 and 30% sucrose solutions (each overnight at 4°C), and embedded in an OCT compound. Coronal cryosections (30 µm thick) were prepared at levels approximately 3–4 mm posterior to the bregma, where the PAG is clearly identifiable. A total of 2–5 sections per mouse were used for analysis.

Free‐floating sections were washed three times for 15 min each in PBS containing 0.3% Triton X‐100, blocked for 1 h in PBS/0.3% Triton X‐100 with 5% normal goat serum, and incubated overnight at 4°C with a rabbit anti‐β‐END antibody (Ab_572220, 1:5000; ImmunoStar, Hudson, WI, USA). Sections were then incubated for 2 h at room temperature with Alexa Fluor 594–conjugated goat anti‐rabbit secondary antibody (A11012, 1:1000; Invitrogen, Thermo Fisher Scientific, Waltham, MA, USA). Images were acquired using a confocal laser scanning microscope (FV10i, Olympus, Tokyo, Japan). To ensure consistent and objective sampling, six fields of view (FOVs) were captured per section at fixed locations around the cerebral aqueduct: three FOVs each on the left and right sides of the PAG. These FOVs were selected strictly based on anatomical landmarks identified using bright‐field images, regardless of β‐END immunofluorescence intensity.

The average number of β‐END–positive signals per field was quantified using ImageJ software (version 1.53k; National Institutes of Health, Bethesda, MD, USA).

### 2.6 mRNA Expression Analysis

Total RNA was extracted from the hypothalamic region including the arcuate nucleus and the PAG tissue using the RNeasy Mini Universal Kit (QIAGEN, Hilden, Germany). RNA concentration and purity were measured using a NanoDrop 2000 spectrophotometer (Thermo Fisher Scientific), and only samples with an A260/280 ratio of 1.8–2.1 were used. Two samples were excluded from *Pomc* mRNA analysis: one mouse in the PBM group died during the housing period, and RNA extraction failed for one sample in the control group. cDNA was synthesized using ReverTra Ace qPCR RT Master Mix (TOYOBO Co., Ltd., Osaka, Japan) according to the manufacturer's instructions and stored at −30°C until use.

Quantitative real‐time PCR (qPCR) was performed using a 7500 Real‐Time PCR System (Applied Biosystems, Foster City, CA, USA) with TaqMan gene expression assays (Thermo Fisher Scientific). The target genes were *Pomc* (Assay ID: Mm00435874_m1) in the hypothalamus and *Oprm1* (Assay ID: Mm01188089_m1) in the PAG, and the reference gene was β‐actin (*Actb*; Assay ID: Mm02619580_g1). Relative expression levels were calculated using the ΔΔCt method.

### 2.7 Statistical Analysis

Data are presented as the mean ± standard error (SE). Group comparisons were performed using the Wilcoxon rank sum test. Additionally, as sexual dimorphism has been reported to influence the effects of various interventions on mechanical withdrawal threshold (Sorge et al. 2015), and MOR expression in the PAG (Loyd et al. 2008), sex was incorporated as a biological variable in our analyses. To evaluate the effects of sex (male vs. female) and intervention (control vs. PBM) on the paw withdrawal threshold ratio at week 3, an analysis of covariance (ANCOVA) was performed. Since baseline threshold ratios differed between sexes (males [*n* = 15], 1.1 ± 0.03; females [*n* = 16], 1.0 ± 0.03; *p* = 0.04, Wilcoxon rank sum test), the baseline ratio was included as a covariate. The effects of sex and intervention on the β‐END immunoreactivity, as well as on *Pomc* and *Oprm1* mRNA expression levels, were analyzed using two‐way analysis of variance (two‐way ANOVA). A *p*‐value < 0.05 was considered statistically significant. Statistical analyses were conducted using JMP Student Edition 18.2.0 (SAS Institute Inc., Cary, NC, USA).

## 3 Results

### 3.1 PBM Increases the Paw Withdrawal Threshold Ratio in Fractured Mice

**FIGURE 3:**
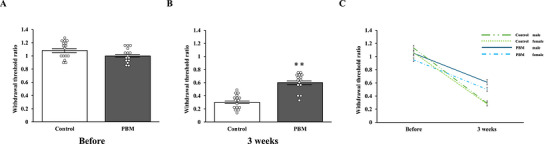
Effects of passive oscillatory body motion (PBM) on paw withdrawal threshold in a murine fracture model. Paw withdrawal threshold ratios (fractured side/contralateral side) were measured using the von Frey filament test before fracture induction and 3 weeks thereafter. (A) No significant difference was observed between groups before fracture induction (PBM: 1.0 ± 0.02; Control: 1.1 ± 0.03; *p* = 0.08); (B) at 3 weeks after fracture induction, the PBM group showed significantly higher threshold ratios compared to the control group (PBM: 0.60 ± 0.03; control: 0.3 ± 0.02; *p* < 0.001). Statistical significance was determined using the Wilcoxon rank sum test (control group: *n* = 16, PBM group: *n* = 15); and (C) ANCOVA revealed no significant interaction between intervention and sex (*p* = 0.44). A significant main effect of intervention was observed, with the PBM group showing higher threshold ratios than the control group (PBM: male, 0.6 ± 0.04; female, 0.5 ± 0.03; control: male, 0.3 ± 0.04; female, 0.3 ± 0.03; main effect of intervention, *p* < 0.001). No significant main effect of sex was detected (*p* = 0.89). ** *p* < 0.001 compared with the control group.

Before fracture induction, there was no significant difference in the paw withdrawal threshold ratio (fractured side/contralateral side) between the PBM group (1.0 ± 0.02) and the control group (1.1 ± 0.03) (*p* = 0.08; Figure 3A). At 3 weeks after fracture induction, the PBM group exhibited a significantly higher threshold ratio (0.6 ± 0.03) compared to the control group (0.3 ± 0.02) (*p* < 0.001; Figure 3B). Consistent with these results, the absolute withdrawal threshold was also significantly increased in the PBM group (Supplementary Figure 3).

The ANCOVA revealed no significant interaction between intervention and sex (*p* = 0.44). A significant main effect of intervention was observed, with the PBM group exhibiting a significantly higher paw withdrawal threshold ratio than the control group (*p* < 0.001). No significant sex differences were observed (*p* = 0.89; Figure 3C).

### 3.2 PBM Enhances β‐END–Positive Signals in the PAG of Fractured Mice

**FIGURE 4:**
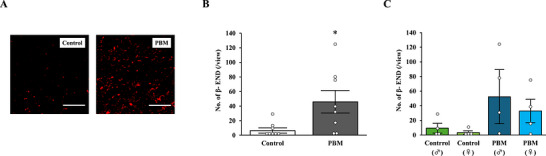
Passive oscillatory body motion (PBM) enhances β‐endorphin (β‐END)–positive signals in the periaqueductal gray (PAG) and is positively associated with the paw withdrawal threshold ratio. (A) Representative confocal images of β‐END immunofluorescence in the PAG from control (left) and PBM‐treated (right) mice. Scale bar = 100 µm; (B) quantification of β‐END–positive signals per field of view (control, *n*  = 8; PBM, *n*  =  8). The PBM group showed significantly higher numbers of β‐END–positive signals per field of view (control: 6.4 ± 3.53; PBM: 45.9 ± 15.29; *p* = 0.02, Wilcoxon rank sum test); (C) two‐way ANOVA revealed no significant interaction between intervention and sex (*p* = 0.54) and no significant main effect of sex on the number of β‐END–positive signals (control‐male: 9.4 ± 6.74, control‐female: 3.3 ± 2.55, PBM‐male: 52.4 ± 36.87, PBM‐female: 32.8 ± 15.99; *p* = 0.33). * *p* < 0.05 compared to the control group.

Immunofluorescence staining in the PAG showed that the average number of β‐END–positive signals per field of view was significantly higher in the PBM group (45.9 ± 15.29) than in the control group (6.4 ± 3.53) (*p* = 0.02; Figures 4A and 4B).

The two‐way ANOVA revealed no significant interaction between intervention and sex (*p* = 0.54). A significant main effect of intervention was observed, with the PBM group exhibiting a higher number of β‐END–positive signals than the control group (*p* = 0.02). No significant sex differences were observed (*p* = 0.33; Figure 4C).

### 3.3 PBM Tends to Increase *Pomc* mRNA Expression in the Hypothalamus of Fractured Mice

**FIGURE 5:**
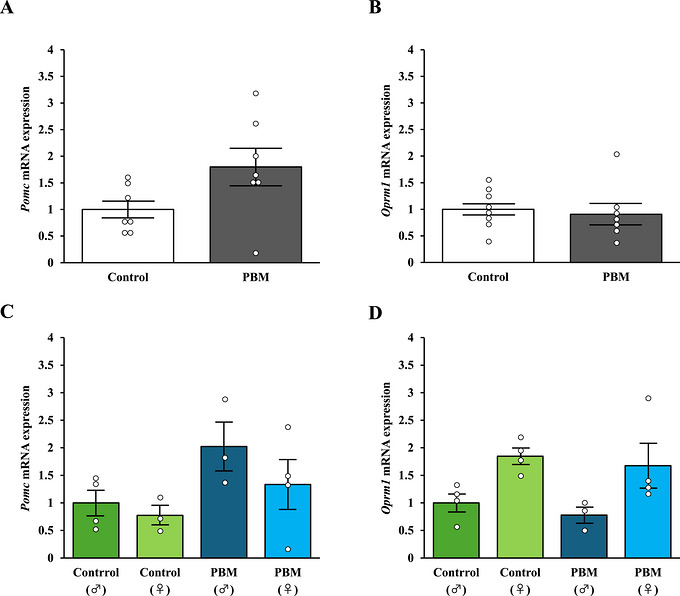
Relative *Pomc* and *Oprm1* mRNA expression after passive oscillatory body motion (PBM) intervention. (A) Relative *Pomc* mRNA expression in the hypothalamus tended to be higher in the PBM group (*n* = 7, 1.8 ± 0.35) than in the control group (*n* = 7, 1.0 ± 0.16), although the difference did not reach statistical significance (*p* = 0.07, Wilcoxon rank sum test); (B) relative *Oprm1* mRNA expression in the PAG did not differ between the PBM group (*n* = 7, 0.9 ± 0.20) and the control group (*n* = 8, 1.0 ± 0.13) (*p* =  0.69, Wilcoxon rank sum test); (C) two‐way ANOVA for hypothalamic *Pomc* mRNA expression showed no significant interaction between intervention and sex (*p* = 0.53). Regarding the main effect of intervention, a trend toward increased expression was observed in the PBM group (*p* = 0.05), while no significant main effect of sex was observed (*p* = 0.23) (control‐male: 1.0 ± 0.23; control‐female: 0.8 ± 0.18; PBM‐male: 2.0 ± 0.44; PBM‐female: 1.3 ± 0.45); and (D) for *Oprm1* mRNA expression in the PAG, two‐way ANOVA revealed no significant interaction (*p* = 0.92) and no significant main effect of intervention (*p* = 0.46), although a main effect of sex was observed (control‐male: 1.0 ± 0.16; control‐female: 1.8 ± 0.15; PBM‐male: 0.7 ± 0.15; PBM‐female: 1.7 ± 0.41; *p* = 0.01).

The qPCR analysis indicated that relative *Pomc* mRNA expression in the hypothalamus tended to be higher in the PBM group (1.8 ± 0.35) than in the control group (1.0 ± 0.16), although this difference was not statistically significance (*p* = 0.07; Figure 5A). Similarly, relative *Oprm1* mRNA expression in the PAG tissue did not differ between the PBM group (0.9 ± 0.20) and the control group (1.0 ± 0.13) (*p* = 0.69; Figure 5B).

For *Pomc* mRNA expression, the two‐way ANOVA revealed no significant interaction between intervention and sex (*p* = 0.53). Regarding the main effect of intervention, the PBM group showed a trend toward increased *Pomc* mRNA expression compared with the control group (*p* = 0.05). No significant sex differences were observed (*p* = 0.23; Figure 5C). In contrast, the two‐way ANOVA for *Oprm1* mRNA expression revealed no significant interaction between intervention and sex (*p* = 0.92), and no significant main effect of intervention was observed (*p* = 0.46). However, a significant main effect of sex was detected, with females showing higher *Oprm1* mRNA expression than males (*p* = 0.01; Figure 5D).

## 4 Discussion

This study demonstrated that PBM alleviated pain‐related behaviors and increased β‐END immunoreactivity in the PAG of mice with fracture pain. These findings suggest that PBM may activate the β‐END–related hypothalamus–PAG pathway, thereby contributing to the improvement of pain‐related behaviors. In addition, qPCR analysis revealed that hypothalamic *Pomc* mRNA expression tended to increase in the PBM group. Since POMC is the precursor of β‐END (Tao et al. 2023), these preliminary data may suggest a potential, though statistically inconclusive, link between PBM and the transcriptional modulation of *Pomc* mRNA. Due to the limited sample size, this transcriptional trend should be interpreted cautiously as a strictly exploratory observation. Collectively, our findings raise the possibility that PBM may exert analgesic effects by facilitating activation of the β‐END–related hypothalamus–PAG pathway. β‐END is synthesize in POMC neurons located in the arcuate nucleus of the hypothalamus (Kang et al. 2021; Tao et al. 2023), and these neurons project their axons to brainstem regions such as the PAG. In the PAG, β‐END is released and contributes to activation of the hypothalamus–PAG pathway (Bagley and Ingram 2020; Ossipov et al. 2014). This pathway consists of multisynaptic inhibitory circuits extending from the PAG to the rostral ventromedial medulla and subsequently to the spinal dorsal horn, a process primarily mediated by MOR (Fields 2004). Consistent with this framework, previous studies have demonstrated that treadmill exercise increases β‐END levels in the PAG and alleviates pain‐related behaviors in rat models of neuropathic pain (Stagg et al. 2011).

PBM, as applied in this study, is a passive vertical linear motion designed to reproduce the vertical accelerations that occur during running, thereby delivering cyclic mechanical stimulation throughout the body without requiring voluntary movement by the animal.

The observation that PBM reduced pain‐related behaviors and enhanced β‐END immunoreactivity suggests that vertical acceleration waveforms, such as those generated during running, may activate central analgesic systems independently of active exercise. However, it should be noted that neuropeptides such as β‐END are synthesized in neurons, transported along axons, and released from nerve terminals. Therefore, immunoreactivity at the terminals reflects the dynamic balance among peptide synthesis, axonal transport, and release. Although an increase in β‐END release induced by PBM could theoretically lead to peptide depletion and thus decreased staining, our results showed a significant increase in β‐END‐positive signals. This finding could reflect either enhanced synthesis and/or axonal transport that exceed the rate of release or, conversely, reduced release and/or delayed transport; however, our data cannot distinguish between these possibilities. Therefore, the precise effect of PBM on β‐END synthesis, axonal transport, and release kinetics remains to be investigated in future studies.

We have previously reported that vertical oscillation influences interstitial fluid dynamics and transmits mechanical stress to neurons in the brain (Ossipov et al. 2014). Based on these findings, it is plausible that the vertical acceleration waveforms induced by PBM similarly influence interstitial fluid dynamics and transmit mechanical stress to hypothalamic POMC neurons. Consequently, POMC neuronal activity may be enhanced, leading to increased release of β‐END. Hypothalamic POMC neurons are known to be activated by treadmill exercise or muscle‐derived cytokines (Kang et al. 2021). Together with these established metabolic pathways, our results suggest that exercise‐induced increases in β‐END may be explained not only by metabolic stimuli but also, at least in part, by mechanical stimulation. Collectively, the vertical acceleration waveforms generated during treadmill running may activate POMC neurons and promote β‐END release, in concert with previously recognized metabolic factors. This study provides the first experimental evidence highlighting the contribution of vertical acceleration waveforms to the mechanisms underlying exercise‐induced analgesia. In contrast, no difference in *Oprm1* mRNA in the PAG was observed between the two groups. A previous study reported that four weeks of treadmill exercise decreased MOR expression in the rostral ventromedial medulla of rats with neuropathic pain (Kim et al. 2015). In the present study, however, the intervention period was limited to three weeks, which may partly explain the absence of detectable changes in *Oprm1* mRNA expression. These findings suggest that longer‐term investigations are warranted. Furthermore, the present study incorporated sex as a biological variable in the analysis. Previous studies have suggested that sex differences may influence pain perception and analgesic efficacy through various interventions, potentially mediated by the effects of gonadal hormones. In particular, the Sex and Gender Equity in Research (SAGER) guidelines recommend that preclinical studies include both male and female animals and the inclusion of sex‐based analyses (Heidari et al. 2016). When this is not feasible, the limitations should be clearly stated. A noteworthy exploratory finding of the present study was that *Oprm1* expression in the PAG tended to be higher in females than in males. In contrast, previous studies have reported higher MOR expression in the ventral PAG of male rats (Loyd et al. 2008). This discrepancy may be attributable to several factors, including the fact that the PAG region analyzed here was not restricted to the ventral portion, the lack of estrous cycle control, and species differences between rats and mice. Additionally, *Oprm1* generates multiple receptor isoforms through alternative splicing, which can vary across brain regions and between sexes (Liu et al. 2018). Therefore, differences in overall *Oprm1* mRNA levels may not necessarily reflect changes in functionally relevant MOR isoforms. It is important to note that the sample size was insufficient for a detailed investigation of sex‐specific effects; thus, our findings regarding sex differences in *Oprm1* mRNA expression should be considered exploratory and hypothesis‐generating. As the analysis included four groups (male and female control groups and male and female PBM groups), resulting in relatively small group sizes (*n* = 3–4 per group). Further validation using a larger sample size is required. In addition, several limitations of this study should be acknowledged. First, our evaluation was limited to β‐END and *Pomc* expression; other endogenous opioids (e.g., enkephalins, dynorphins) and non‐opioid analgesic pathways (e.g., cannabinoids, monoaminergic systems) were not examined. Future studies should adopt a more comprehensive molecular approach. Second, the oscillation parameters of PBM (frequency and amplitude) were restricted to a single setting; thus, the effects of varying stimulation conditions remain to be investigated. Third, we did not monitor spontaneous activity during the housing period, which may have influenced pain‐related behaviors or endogenous analgesic mechanisms. Furthermore, although we have previously demonstrated interstitial fluid movement in the cerebral cortex and brainstem (Murase et al. 2023; Ryu et al. 2020), the dynamics of interstitial fluid within the hypothalamus and PAG during PBM have not yet been directly measured. While it is plausible that similar hydrodynamic changes occur in these regions, this remains hypothetical, and future studies should aim to visualize and quantify these dynamics. Fourth, we did not compare the effects of PBM with those of treadmill exercise, which is known to induce β‐END expression. Fifth, as the efficacy of PBM was evaluated solely in a murine fracture model, further research is required to investigate its effects in other pain models, such as neuropathic pain and nociplastic pain models. Sixth, the von Frey hair test was not performed under blinded conditions, as the experimenter was aware of group allocations; therefore, the possibility of observer bias cannot be ruled out. Seventh, the experiment assessing restraint stress (Supplementary Figure 2) had a small sample size (*n* = 5 per group) and minor protocol variations from the main study. Therefore, its null result (*p* = 0.40) may be limited by statistical power, and the possibility that restraint stress affects pain‐related behavior and the opioid system cannot be entirely excluded. Finally, we did not perform histological or imaging assessments of fracture healing and local inflammation, both of which may also influence central mechanisms of pain regulation.

## 5 Conclusions

In this preliminary study, we provide evidence that PBM, a form of externally applied vertical mechanical stimulation, alleviates pain‐related behaviors in a murine fracture model. PBM increased β‐END immunoreactivity in the PAG, suggesting activation of the endogenous opioid system. These findings indicate that PBM serves as a non‐invasive intervention capable of activating the β‐END–related hypothalamus–PAG pathway without requiring voluntary physical activity. PBM may represent a potential therapeutic option for patients who have difficulty engaging in active exercise.

## Author Contributions

**Kazuhiro Hayashi**: methodology, formal analysis, Writing – review and editing. **Ryoma Kato**: conceptualization, investigation, data curation, writing – original draft, formal analysis. **Yuri Okada**: methodology, formal analysis, writing – review and editing. **Yasuhiro Sawada**: methodology, formal analysis, writing – review and editing. **Rina Okura**: methodology, formal analysis, writing – review and editing. **Fuyu Niida**: methodology, formal analysis, writing – review and editing. **Naoyoshi Sakitani**: methodology, formal analysis, writing – review and editing. **Akira Ito**: conceptualization, supervision, funding acquisition, writing – review and editing. **Jun Miyata**: methodology, formal analysis, writing – review and editing.

## Ethics Statement

All experimental procedures were approved by the Animal Research Committee of Kyoto University (Approval No. MedKyo24557).

## Conflicts of Interest

The authors declare no conflicts of interest.

## Declaration of Generative AI and AI‐Assisted Technologies

During the preparation of this work, the authors used ChatGPT and Gemini 3 to assist with preparing an initial draft, language polishing, and translation. After using these tools, the authors carefully reviewed and edited the content as needed and take full responsibility for the accuracy and integrity of the final manuscript.

## Supporting information




**Supplementary Material**: brb371614‐sup‐0001‐FigureS1‐S3.docx

## Data Availability

The data that support the findings of this study are available from the corresponding author upon reasonable request.
