# Supplementary material for: Passive Oscillatory Body Motion Attenuates Pain‐Related Behaviors and Increases β‐Endorphin Immunoreactivity in a Murine Fracture Model: A Preliminary Study
Source: Brain Behav. 2026 Jul 23;16(7):e71614. doi: 10.1002/brb3.71614 (PMC13396874; doi:10.1002/brb3.71614)
Supplement: Supplementary file 1 — Supplementary Material: brb371614‐sup‐0001‐FigureS1‐S3.docx [file BRB3-16-e71614-s001.docx]

**Supplementary data**

**Supplementary Figure 1. Verification of the reproducibility of the manual tibial fracture procedure.**
Male C57BL/6J mice (14–15 weeks old) were used, and a manual closed tibial fracture was created in both hind limbs of each animal under anesthesia. The fracture procedure was performed by a single experienced operator to ensure consistency. Immediately following fracture induction, mice were euthanized, and the distal hind limbs were surgically exposed for macroscopic examination. The photograph shows a representative macroscopic image of the distal lower leg following dissection and removal of soft tissues. The fractured tibiae were examined to confirm the absence of additional (third) bone fragments. Additionaly, the distance from the plantar surface to the fracture site (arrow) was measured. No third bone fragments were observed in any hind limbs, and the distance from the plantar surface to the fracture site ranged from 6 to 9 mm, confirming the reproducibility of the fracture procedure.

**Supplementary Figure 2. Effects of mouse restraint on pain-related behavior.**
To examine the impact of restraint stress on pain-related behavior, a murine fracture model was established using 7-week-old male C57BL/6J mice. Following right tibial fracture, the right hind limb was immobilized with a cast and tape for three weeks. Mice were randomly assigned to two groups: a fracture + cast fixation group (Control; n = 5) and a fracture + cast fixation plus restraint stress group (Restraint Stress; n = 5). Restraint stress was applied under awake conditions for 30 min per day, 5 days per week, for three consecutive weeks starting the day after fracture induction. After three weeks, the cast was removed, and mechanical withdrawal thresholds were measured in both the fractured and contralateral hind paws using the von Frey filament test. The ratio of the withdrawal threshold of the fractured paw to that of the contralateral paw (threshold ratio) was used as an index of pain sensitivity. Before model induction, the threshold ratio was 1.1 ± 0.05 in the fracture + cast fixation group and 1.0 ± 0.10 in the fracture + cast fixation + restraint stress group (p = 0.40; Wilcoxon rank sum test). Three weeks post-induction, the threshold ratios were 0.3 ± 0.05 and 0.4 ± 0.04, respectively (p = 0.38; Wilcoxon rank sum test). No significant differences were observed between groups at any time point, indicating that restraint procedure did not significantly affect pain-related behavior.

**Supplementary Figure 3. Absolute withdrawal thresholds of the fractured hind limb.**

Absolute paw withdrawal thresholds (g) of the fractured (left) hind limb in the control group (n = 16; 1.35 ± 0.12) and PBM group (n = 15; 2.4 ± 0.16). Consistent with the threshold ratio results presented in the main text, the PBM group exhibited significantly higher absolute thresholds compared to the control group, confirming the analgesic effect of PBM. ** p < 0.001 compared to the control group (Wilcoxon rank sum test).
